# Fracture du pancréas post traumatique

**DOI:** 10.11604/pamj.2015.20.35.5795

**Published:** 2015-01-14

**Authors:** Addourouj Mohamed Ilyass, Yassine Ibrahimi

**Affiliations:** 1 Service des Urgences Chirurgicales Viscérales, Centre Hospitalier Ibn Sina, Rabat, Maroc

**Keywords:** Traumatisme, pancréas, chirurgie, Trauma, pancreas, surgery

## Image en medicine

Les traumatismes du pancréas sont rares. Ils constituent 0.2 à 6% de l'ensemble des traumatismes de l'abdomen. Le traumatisme isolé du pancréas est peu fréquent et de diagnostic souvent difficile Nous rapportant le cas d'un patient de 20 ans maçon de profession qui a été admis aux urgences pour prise en charge des épigastralgies secondaires a un traumatisme abdominale fermé du a un coup accidentel par un bâton de fer datant de plus de 48 heures. L'abdomen était sensible, la lipasémie était à 10 fois la normale. La TDM montrait une fracture isthmique du pancréas avec un épanchement abdominale de moyenne abondance. Le patient a été admis en urgence au bloc opératoire; il a bénéficie d'une anastomose pancréaticojéjunale; l’évolution été favorable. La rareté des Traumatismes isolés du pancréas et leur symptomatologies cliniques atypiques au stade de début rend leur diagnostic relativement difficile d'autant plus que la biologie n'est pas spécifique. Ceci doit nous pousser à réaliser des TDM devant tout traumatisme épigastrique même s'il n'est pas violent.

**Figure 1:**
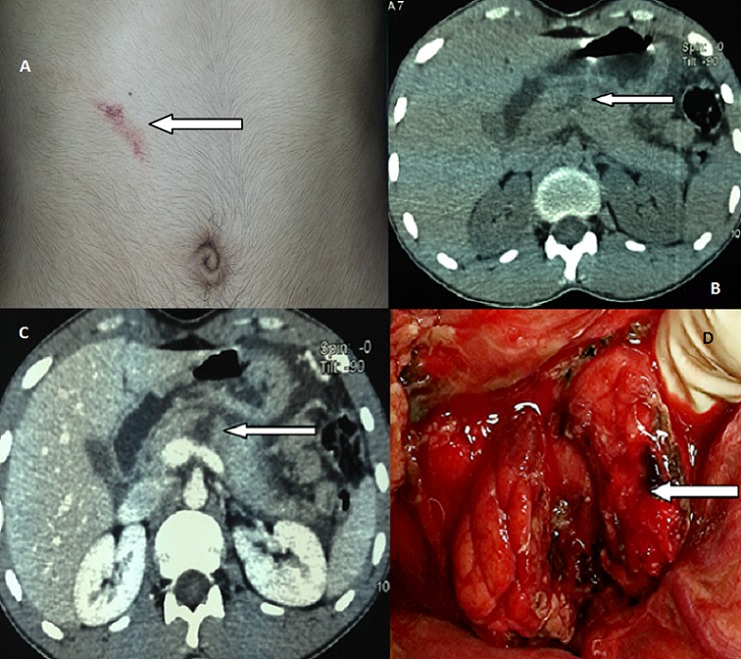
A) siège du traumatisme abdominale Clinical picture showing the seat of abdominal trauma; B) tomodensitometries montrant une fracture isthmique du pancreas CT image showing a fracture of the isthmus pancreas; C) tomodensitometriques montrant une fracture isthmique du pancreas CT image showing a fracture of the isthmus pancreas; D) Image per opératoire montrant une fracture pancréatique Intraoperative picture showing a pancreatic fracture

